# *mixIndependR*: a R package for statistical independence testing of loci in database of multi-locus genotypes

**DOI:** 10.1186/s12859-020-03945-0

**Published:** 2021-01-06

**Authors:** Bing Song, August E. Woerner, John Planz

**Affiliations:** Department of Microbiology, Immunology and Genetics, University of North Texas Health Science Center, 3500 Camp Bowie Blvd, Fort Worth, TX 76107 USA

**Keywords:** Linkage disequilibrium, R package, Non-random association, Mutual independence, STRs, SNPs

## Abstract

**Background:**

Multi-locus genotype data are widely used in population genetics and disease studies. In evaluating the utility of multi-locus data, the independence of markers is commonly considered in many genomic assessments. Generally, pairwise non-random associations are tested by linkage disequilibrium; however, the dependence of one panel might be triplet, quartet, or other. Therefore, a compatible and user-friendly software is necessary for testing and assessing the global linkage disequilibrium among mixed genetic data.

**Results:**

This study describes a software package for testing the mutual independence of mixed genetic datasets. Mutual independence is defined as no non-random associations among all subsets of the tested panel. The new R package “*mixIndependR*” calculates basic genetic parameters like allele frequency, genotype frequency, heterozygosity, Hardy–Weinberg equilibrium, and linkage disequilibrium (LD) by mutual independence from population data, regardless of the type of markers, such as simple nucleotide polymorphisms, short tandem repeats, insertions and deletions, and any other genetic markers. A novel method of assessing the dependence of mixed genetic panels is developed in this study and functionally analyzed in the software package. By comparing the observed distribution of two common summary statistics (the number of heterozygous loci [K] and the number of share alleles [X]) with their expected distributions under the assumption of mutual independence, the overall independence is tested.

**Conclusion:**

The package “*mixIndependR*” is compatible to all categories of genetic markers and detects the overall non-random associations. Compared to pairwise disequilibrium, the approach described herein tends to have higher power, especially when number of markers is large. With this package, more multi-functional or stronger genetic panels can be developed, like mixed panels with different kinds of markers. In population genetics, the package “*mixIndependR*” makes it possible to discover more about admixture of populations, natural selection, genetic drift, and population demographics, as a more powerful method of detecting LD. Moreover, this new approach can optimize variants selection in disease studies and contribute to panel combination for treatments in multimorbidity. Application of this approach in real data is expected in the future, and this might bring a leap in the field of genetic technology.

**Availability:**

The R package *mixIndependR,* is available on the Comprehensive R Archive Network (CRAN) at: https://cran.r-project.org/web/packages/mixIndependR/index.html.

## Background

Genetic polymorphisms are commonly classified into different types, such as SNPs, InDels, and STRs. Different characteristics, such as mutation rates, deviations, and heterozygosity, make those markers isolated in application. However, due to their specific advantages, such as the higher power of discrimination of STRs, and the important roles of SNPs in application of ancestry informative markers (AIMs) and prediction of physical traits [1], mixed multiplex assays are considered more multi-functional [2–4] in some applications of molecular genetics. While there are many studies that compare one marker to others of their kind (SNPs to SNPs, STRs to STRS), only rarely are comparisons made between marker classes. A few notable exceptions include a 2002 study that noted the linkage disequilibrium (LD) of SNPs and microsatellites [5]. Later in 2017, 13 CODIS STR markers were found to be matched to genome-wide SNP profiles with median accuracies in excess of 90% [4]. Even though the disequilibrium among different markers can be significant and the hypothesis of independence may require testing, most of the software available now is designed specifically for one kind of those markers. For example, *adegenet,* which analyzes SNPs and STRs separately; *BCFTools* [6] and *SNPrelate* [7]*,* both of which focus on SNPs; and Genetic Data Analysis (GDA) [8], which targets at STRs specially. Therefore, a software compatible with multi-type genetic markers simultaneously that can test independence between different type of data is needed.

Another shortcoming of traditional measures of LD is the way it is assessed, that is to test pairwise linkage disequilibrium. However, due to more accurate conception, non-random associations can be not only pairwise associations, but also triplet, quartet, and higher order associations among all sites, defined as mutual independence. D′ is sometimes used to assess the higher-level associations, but it is time consuming to obtain due to numerous calculations. Therefore, summary statistics become a choice to avoid all these excess and burden.

A new package, *mixIndependR*, was developed for testing mutual independence among all sites of the multi-locus genotypes mixed type of genetic markers for the R software. As a larger category of dependency across all over the panel needs to be tested, summary statistics, which are able to give a quick and comprehensive description of the dataset, came into consideration. In an early study [9, 10], summary statistics, such as the number of heterozygous loci (K) and the number of shared alleles (X), were used to test the significance of disequilibrium (non-random associations) or dependency between loci by comparing the observed and expected distributions under the hypothesis of mutual independence. Functions for obtaining basic genetic parameters like allele frequency, genotype frequency, heterozygosity, and Hardy–Weinberg equilibrium (HWE) are also included in *mixIndependR* to increase its versatility.

## Results

### Software pipeline

This section presents how a multi-locus genotype panel with mixed genetic markers (SNPs and STR) can be tested for its mutual independence using *mixIndependR*. In the following example, a sample with 6 STRs and 94 SNPs (Additional file 8: Table S1) was tested using *mixIndependR*. This panel was designed after variant selection and pruning with the threshold $r^{2} < 0.1$ by *plink (version 1.90)*, based on the genotypes and the reference panel created by Saini et al. [11]. This reference haplotype panel was created by imputing STR genotypes into existing SNPs datasets. The STRs were selected from genome-wide catalog of STR variation they generated in Simons Simplex Collection (SSC) cohort, and genotype data for 2504 individuals are from existing 1000 genome phase 3 [11]. The threshold for independent panels is usually set as 0.2 [12, 13], but here we narrowed the scope in pruning to get a panel with less possibility to carry dependency. In addition, pairwise correlations of this dataset were exported and check, and no dependent pairs have been found with the usual threshold of 0.2. This dataset is included in the *mixIndependR* package under the name “mixexample”. *mixIndependR* is compatible with genotype files in a tabular format (e.g., excel, cvs) and the variant call format (vcf) [14]. The allele separator in the genotypes needs to be specified using the “sep” parameter.

- x <- mixexample # This panel with 96 SNPs and 4 STRs are filtered from a reference haplotype panel developed by Saini et al. [11]

*mixIndependR* can be used to estimate basic genetic parameters, such as allele frequencies, tables of heterozygosity and of shared alleles, and to test Hardy Weinberg equilibrium in either Pearson’s.

#### Chi-squared test.

- p <- **AlleleFreq**(x,sep = "\\|") # calculate the table of allele frequencies and return a table with rows of alleles and columns of markers (Additional file 9: Table S2).
- G <- **GenotypeFreq**(x,sep = "\\|",expect = FALSE) # calculate the observed genotype frequencies with genotypes in rows and markers in columns.
- G0 <- **GenotypeFreq**(x,sep = "\\|",expect = TRUE) # calculate the expected genotype frequencies under Hardy–Weinberg equilibrium (HWE).
- h <- **Heterozygous**(x,sep = "\\|") # obtain the table of heterozygosity with rows of individuals and columns of markers, where “1” denotes heterozygous while “0” denotes homozygous (Additional file 10: Table S3).
- H <- **RxpHetero**(h,p,HWE = TRUE) # calculate the observed average heterozygosity or the expected heterozygosity on each marker under the assumption of HWE.
- AS <- **AlleleShare**(x,sep = "\\|",replacement = FALSE) # calculate the table of shared alleles of each pair of individuals for each marker with the rows denoting the pairs of individuals and the columns denoting the variants. If the replacement is TRUE, the pairs are formed with replacement randomly and this table includes all possible pairs; if the replacement is FALSE, the pairs are formed without replacement randomly and the number of pairs equals half of the sample size. (Additional file 11: Table S4).
- e <- **RealProAlleleShare**(AS) # calculate the observed proportions of sharing 0, 1 or 2 alleles on each marker.
- e0 <- **ExpProAlleleShare**(p) # calculate the expected probabilities of sharing 0, 1 or 2 alleles on each marker for any unrelated individuals. This process is according to the functions described by Weirs et al. [15]
- HWE_pvalue <- **HWE.Chisq**(G,G0,rescale.p = T, simulate.p.value = T,B = 2000) # test the HWE with Pearson’s Chi-square test and export the *p* value for each marker.

According to the mathematical principles described in previous studies of Chakraborty et al. [9, 10], the observed and expected distribution of the number of heterozygous loci (K) and number of share alleles (X) can be constructed and visualized (Additional file 1: Figure S1, Additional file 2: Figure S2).

- ObsDist_K <- **FreqHetero**(h) # calculate the observed distribution of K, exporting the frequencies of K at the values from 0 to total number of tested markers.
- ExpDist_K <- **DistHetero**(H) # calculate the expected distribution of K, exporting the probabilities of K at the values from 0 to total number of tested markers.
- ObsDist_X <- **FreqAlleleShare**(AS) # calculate the observed distribution of X, exporting the frequencies of X at the values from 0 to 2 times of total number of tested markers.
- ExpDist_X <- **DistAlleleShare**(e) # calculate the expected distribution of X, exporting the probabilities of X at the values from 0 to 2 times of total number of tested markers.
- df_K <- **ComposPare_K**(h,ExpDist_K,trans = F) #generate comparison data frame for observed and expected distributions. If “trans = F”, the table contains a variable “OvE” denoting the category of “observed” or “expected”, and a variable “Freq” denoting the frequencies. This format is prepared for visualization in “ggplot2”. If “trans = T”, the result is made by two columns- “K_io” and “K_ie” which denoting the observed and expected K for each individual, and the number of rows equals to the number of individuals of the sample.
- df_X <- **ComposPare_X**(AS,ExpDist_X,trans = F) #generate comparison data frame for observed and expected distributions. If “trans = F”, the table contains a variable “OvE” denoting the category of “observed” or “expected”, and a variable “Freq” denoting the frequencies. This format is prepared for visualization in “ggplot2”. If “trans = T”, the result is made by two columns- “X_io” and “X_ie” which denoting the observed and expected X for each pair of individuals, and the number of rows equals to the number of pair of individuals of table of number of shared alleles AS.

Not only were the visual comparisons made, but statistical tests were also conducted using this package. Considering the expected distributions under assumption of mutual independence follow restricted multinomial distributions with fixed probabilities on each trial, no traditional statistic tests can be applied (e.g. traditional chi-square test for multinomial distribution). Instead, simulations were conducted to obtain null distributions of chi-square values and cumulative probability functions were built to find an accurate critical value.

- Simu_K <- **Simulate_DistK**(H,2504,500) #build 500 times of simulations for distribution of K with the sample size equals to the original data for each simulation, under the mutual independence assumption (Additional file 12: Table S5).
- Simu_X <- **Simulate_DistX**(e,1252,500) #build 500 times of simulations for distribution of X with the sample size equals to the number of pairs composed in “AS” table for each simulation, under the mutual independence assumption (Additional file 13: Table S6).
- x2_K <- **Dist_SimuChisq**(Simu_K,ExpDist_K$Density,1000) # Calculate 500 Chi-square values for K with 1000 times of replicates in each and export the set of *p* values.
- x2_X <- **Dist_SimuChisq**(Simu_X,ExpDist_X$Density,1000) # Calculate 500 Chi-square values for X with 1000 times of replicates in each and export the set of *p* values.
- P1 <- **ecdf**(x2_K) #Build the cumulative probability of function for the set of Chi-square values. Note: this is the function in the package of “stats”.
- P2 <- **ecdf**(x2_X) #Build the cumulative probability of function for the set of Chi-square values. Note: this is the function in the package of “stats”.

If the significant level is 0.05, the critical value of the chi-square value for each specific expected distribution is the value when cumulative probability equals 95%. In Additional file 3: Figure S3 and Additional file 4: Figure S4, the cross points of vertical and horizontal line are the critical values. In this example, the critical value for K is 112.9 and the test chi-square value equals 77.417; while 115.0 as critical value for X and test statistic is 62.229. In both statistics, we fail to reject the null hypothesis and may conclude the panel is mutual independent. This results also agree with the selection rules in the process of variant pruning on plink that the pairwise squared correlation between genotype allele counts “--indep-pairwise” is smaller than 0.1 [16, 17]. However, the result in this section represents a single analysis, and the results can vary among different trials.

### Statistical analysis

**Fig. 1:**
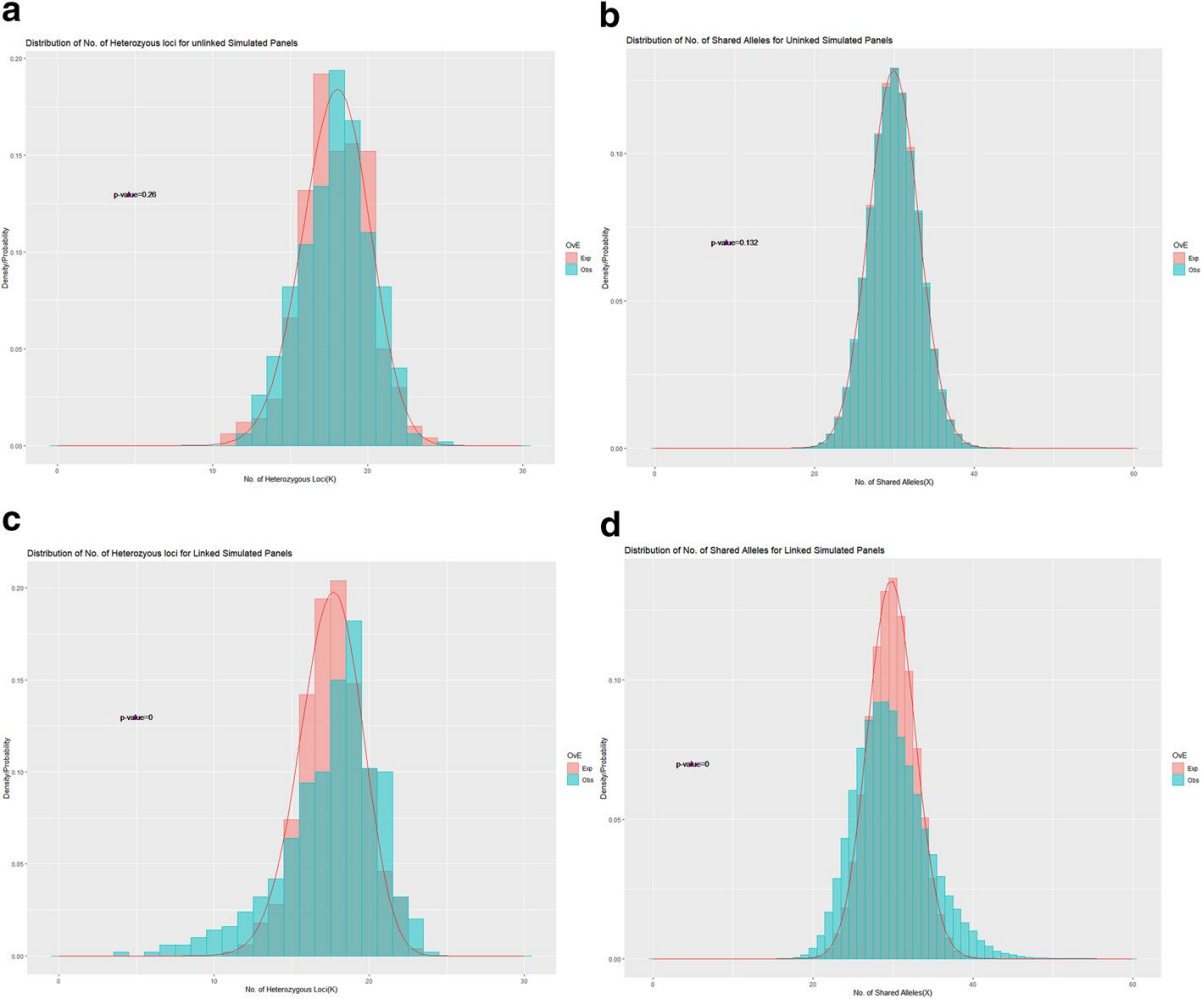
Distribution of number of heterozygous loci and shared alleles for unlinked vs fully linked data. The simulated dataset is a mixed genetic panel with 20 STRs and 10 SNPs for 500 random individuals. **a**, **b** are the distribution comparisons of number of heterozygous loci and Shared Alleles (non-overlapped pairs) for unlinked data. All STRs and SNPs were generated from different chromosomes randomly. **c**, **d** are distribution comparisons of number of heterozygous loci and Shared Alleles (non-overlapped pairs) for linked data. All STRs were from the same chromosome, and all SNPs were from the same chromosome. However, STRs and SNPs were randomly grouped. Red line in plots are curves of expected probabilities

**Fig. 2:**
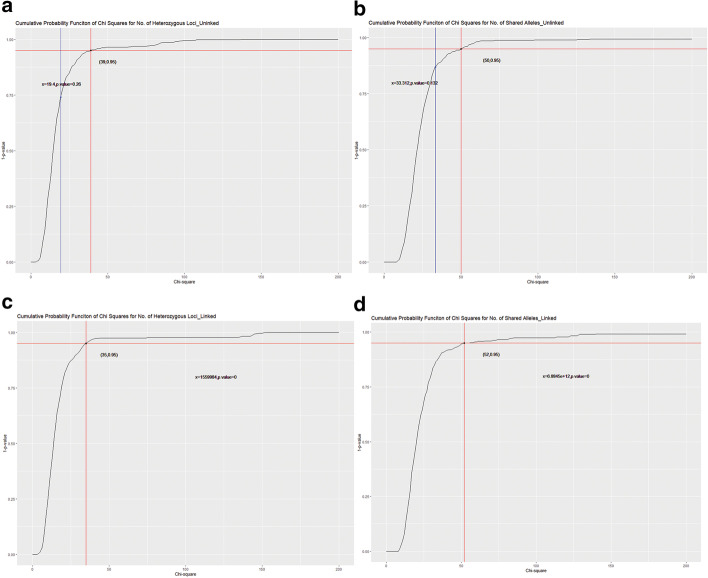
Curve of cumulative probability of Chi-square values for number of heterozygous loci or shared alleles. Red line is where the critical value lies for the confidence level of 95%; while the blue one is the test statistic. **a**, **b** are figures for unlinked data; **c**, **d** are figures for fully linked data. In (**c**) and (**d**), the test statistic is far larger than the critical value, and beyond the limit of x-axis

Both simulation and real data have been tested with this approach. We simulated unlinked and linked data under the Wright-Fisher neutral model using the software *ms* [18]; the microsatellite data were converted by *microsat* [19] from *ms* format. In the single-comparison example, we used the simulated panel with 20 STRs and 10 SNPs for unlinked and fully-linked scenarios and the sample size is 500 in each case. The comparisons of the single-run for unlinked data and fully-linked data were made and presented in Figs. 1 and 2.

Consistent with expectations, the unlinked STR-SNP panel shows significant difference neither for K (*p* value = 0.26) (Fig. 1a) or X (*p* value = 0.13) (Fig. 1b). At the same time, significant LD is detected for both of K (*p* value < 0.0001) and X (*p* value < 0.0001) at the fully linked STR-SNP panels (Fig. 1c, d). It is contrastive that, for the simulated unlinked panel, the observed distribution of K and X matched the expected one visually under the assumption of mutual independence while, for the fully-linked panel, there is significant offset between the observed and expected distributions.

For the unlinked panel, the test statistic of *K* is 19.40 and that of *X* is 33.31, both smaller than the critical value 39.00 and 50.00, respectively (Fig. 2a, b). However, the test values (1,559,984 and 6.8834 × 10^12^ for *K* and *X,* respectively) for the fully linked panel are much larger than the critical values (35.00, 52.00 for *K* and *X,* respectively) (Fig. 2c, d).

Simulated data with different levels of linkage were tested to determine the type I error and power of the method. Figures 1 and 2 presents the results for completely unlinked and fully linked panels (20 STRs and 10 SNPs). To test different extents of LD more adequately, different numbers of random selected genetic unlinked and fully linked markers were imported to the package and tested for mutual independence. *p*-value, which equals to one minus the cumulative probability was chosen as the statistic. Among the 1000 in silico replicates, the percentages of significant cases are recorded in Table 2 for different levels of linkage. In this process, mixed panels made up with equal numbers of SNPs and STRs were simulated, and each level of linkage was set up in three ways: biased to SNPs, biased to STRs, and unbiased. For example, in a panel of 20 markers, 10 SNPs and 10 STRs were selected; if the linkage is set as half-linked in the SNP-biased panel, there would be 10 linked SNPs and 10 unlinked STRs. In contrast, if the panel is STR-biased, 10 linked STRs and 10 unlinked SNPs would be chosen. In the unbiased group, the linked STRs and linked SNPs are always of the same number, which is 5 linked SNPs, 5 linked STRs, 5 unlinked SNPs, and 5 unlinked STRs in the unbiased half-linked panels of 20 markers (Details in Tables 1, 2 and Additional file 14: Table S7).

**Table 1:** The rules of panel design for power test of simulation

Panel size	Number of linked markers	Number of linked SNPs	Number of linked STRs	Linkage	Bias
x	0	0	0	Unlinked	Both
x	2	2	0	OnePair-Linked	SNP
x	2	0	2	OnePair-Linked	STR
x	0.125x	0.125x	0	HalfQuarter-Linked	SNP
x	0.125x	0	0.125x	HalfQuarter-Linked	STR
x	0.125x	0.0625x	0.0625x	HalfQuarter-Linked	Both
x	0.25x	0.25x	0	Quarter-Linked	SNP
x	0.25x	0	0.25x	Quarter-Linked	STR
x	0.25x	0.125x	0.125x	Quarter-Linked	Both
x	0.5x	0.5x	0	Half-Linked	SNP
x	0.5x	0	0.5x	Half-Linked	STR
x	0.5x	0.25x	0.25x	Half-Linked	Both
x	0.75x	0.5x	0.25x	ThreeQuarter-Linked	SNP
x	0.75x	0.25x	0.5x	ThreeQuarter-Linked	STR
x	0.75x	0.375x	0.375x	ThreeQuarter-Linked	Both
x	x-2	0.5x	0.5x-2	Almost-Linked	SNP
x	x-2	0.5x-2	0.5x	Almost-Linked	STR
x	x	0.5x	0.5x	Fully-Linked	Both

The “PanelSize” is the number of markers in each panel and in each panel, half markers are SNPs and the other half markers are STRs. In this table to illustrate the rules of design, “PanelSize” is set to “x”

The variable “Linkage” is defined as the percentage of linked markers among the total. For example, if the “PanelSize” is x, the number of linked markers is 0.5x, then the “Linkage” equals to “Half-Linked”. The “OnePair-Linked” and “Almost-Linked” have unfixed percentage of linked markers but hold the numbers of linked or unlined markers. If there are only 2 linked markers, the “Linkage” is defined as “OnePair-Linked”; if there are only 2 unlinked markers and all others are linked, the “Linkage” is defined as “Almost-Linked”

The variable “Bias” is defined as the distribution of linked markers. If the number of linked SNPs is larger than linked of STRs, the “Bias” equals to “SNP”; in contrast, if the number of linked SNPs is smaller than linked STRs, the “Bias” equals to “STR”; but if the number of linked SNPs and STRs are the same, “Bias = Both”. When the linked markers are less than 50% of total markers, in the biased groups, all linked markers are the same type of markers- the biased one. When the linkage is higher than “Half-Linked”, all the biased markers are linked, and the unlinked markers are found on the other type

**Table 2:** Proportion of *p* values < 0.05 in 1000 times for different levels of linkage using summary statistics (only “Bias = Both” is shown, more details in Additional file 14: Table S7)

X.1	Panel size	Number of linked markers	Linkage	Bias	Total number of heterozygous loci (K)	Total number of shared alleles (X)
1	10	0	Unlinked	Both	0.047	0.039
2	20	0	Unlinked	Both	0.05	0.036
3	30	0	Unlinked	Both	0.051	0.048
4	40	0	Unlinked	Both	0.055	0.038
5	50	0	Unlinked	Both	0.045	0.053
6	100	0	Unlinked	Both	0.047	0.051
31	16	2	HalfQuarter-Linked	Both	0.045	0.044
32	32	4	HalfQuarter-Linked	Both	0.069	0.041
33	48	6	HalfQuarter-Linked	Both	0.072	0.063
34	64	8	HalfQuarter-Linked	Both	0.082	0.065
35	80	10	HalfQuarter-Linked	Both	0.094	0.068
36	96	12	HalfQuarter-Linked	Both	0.109	0.069
53	8	2	Quarter-Linked	Both	0.015	0.047
54	16	4	Quarter-Linked	Both	0.03	0.058
55	24	6	Quarter-Linked	Both	0.057	0.074
56	32	8	Quarter-Linked	Both	0.052	0.094
57	40	10	Quarter-Linked	Both	0.186	0.09
58	64	16	Quarter-Linked	Both	0.303	0.137
59	80	20	Quarter-Linked	Both	0.382	0.154
60	96	24	Quarter-Linked	Both	0.421	0.209
81	8	4	Half-Linked	Both	0.011	0.116
82	12	6	Half-Linked	Both	0.29	0.15
83	16	8	Half-Linked	Both	0.326	0.171
84	20	10	Half-Linked	Both	0.388	0.221
85	24	12	Half-Linked	Both	0.419	0.242
86	32	16	Half-Linked	Both	0.513	0.297
87	40	20	Half-Linked	Both	0.638	0.374
88	60	30	Half-Linked	Both	0.83	0.516
89	80	40	Half-Linked	Both	0.934	0.687
90	100	50	Half-Linked	Both	0.982	0.819
105	16	12	ThreeQuarter-Linked	Both	0.582	0.372
106	24	18	ThreeQuarter-Linked	Both	0.738	0.499
107	32	24	ThreeQuarter-Linked	Both	0.845	0.635
108	40	30	ThreeQuarter-Linked	Both	0.914	0.719
109	64	48	ThreeQuarter-Linked	Both	0.986	0.943
110	80	60	ThreeQuarter-Linked	Both	0.997	0.974
111	96	72	ThreeQuarter-Linked	Both	0.999	0.995
124	10	10	Fully-Linked	Both	0.604	0.404
125	20	20	Fully-Linked	Both	0.835	0.691
126	30	30	Fully-Linked	Both	0.957	0.849
127	40	40	Fully-Linked	Both	0.989	0.936
128	50	50	Fully-Linked	Both	0.997	0.973
129	100	100	Fully-Linked	Both	1	1

For both number of heterozygous loci and number of shared alleles, the proportions of detecting dependency in unlinked dataset are approximately less than 5%; in fully linked dataset, is over 70% when the number of markers is larger than 10. When the percentage of linked markers increases, the power of test increases

For the unlinked panels, the proportion of *p* values smaller than 0.05 was approximately 50 out of 1000 times. As the number of markers increased, the proportions of significant results became closer to 0.05 (Table 2 and Additional file 14: Table S7). The significant cases in the unlink panel are the Type I errors, which indicates the significance level is about 0.05 for this method. In a fully linked dataset, the power of this test was over 50% when one panel had more than 20 markers, and as the sizes of panels increase, the power of K and X is stronger and stronger.

**Fig. 3:**
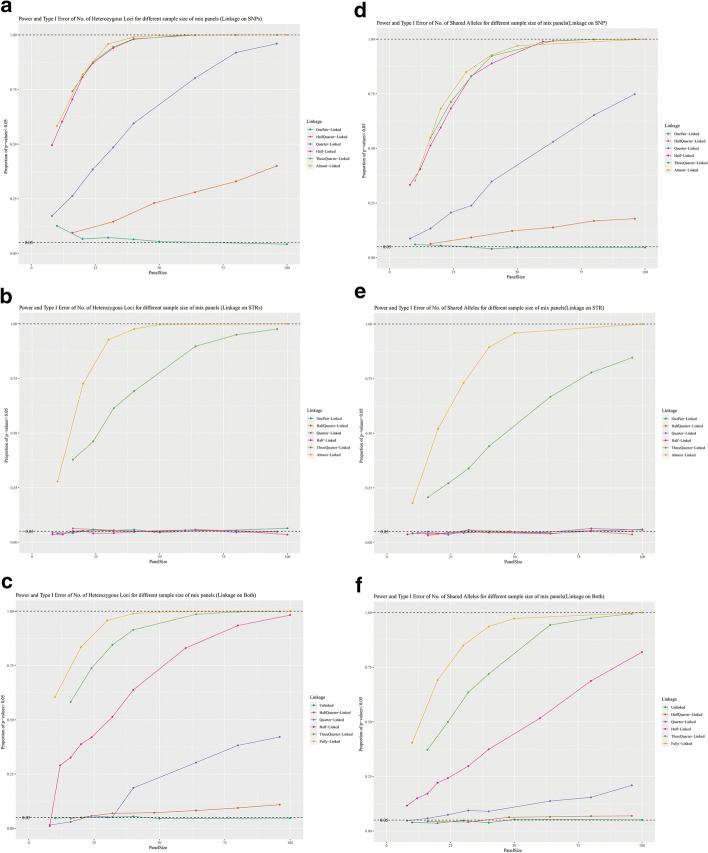
Power and significance level (proportion of *p* values < 0.05) for different levels of linkage. X-axis denotes the number of markers in one panel; Y-axis denotes the proportion of cases when *p* values < 0.05 out of 1000 cases. For the completely unlinked panels, this proportion means significance level (Type I error) in null hypothesis; for other panels with linkages, this proportion means power (1- type II error) of these two methods. **a**–**c** are figures of K and **d**–**f** are figures of X. **a**, **d** are panels with all linked markers are SNPs; **b**, **e** are panels with all linked markers on STRs; **c**, **f** are panels with equal number of linked SNPs and STRs. In condition, power increases with panel size extends; linkage on SNPs contributes more power than STRs; and K shows more power than X. For SNP-biased (linkage on SNPs) panels, dependency can be detected when linkage is quarter-linked or more; but for STR-biased panels, only three-quarter-linked and almost -linked panels can be tested as dependent panels. In unbiased panels, half-quarter-linkage are also hardly to be detected

As the visualization of Table 2 and Additional file 14: Table S7, comparison of different levels of linked data is presented in Fig. 3 (Fig. 3). In addition, three linkage bias scenarios were also compared at each level of linkage (Additional file 5: Figure S5), and the comparisons of K and X are shown in Additional file 6: Figure S6.

The power of *mixIndependR* appears to follow three basic trends: (1) when the number of markers increases, the power of testing increases (Fig. 3); (2) compared to STRs, linkage in SNPs gives more power for linkage tests (Additional file 5: Figure S5); (3) between the two summary statistics, the number of heterozygous loci (K) can detect dependency more often than the number of shared alleles (X) for the same panel (Additional file 6: Figure S6).

When there is only one pair of genetic markers linked, neither of the two methods detected the dependency effectively. In half-quarter-linked panels, the SNP-biased group has a little higher power than STR-biased group- around 10–40% on K and 6% to 18% on X; and power of the unbiased group is between SNP-biased and STR-biased groups, but none of the three groups present a strong enough power in half-quarter-linked panels. However, for quarter- or higher-levels of linkage both K and X yield power greater than 50% when the number of parkers is no less than 60 in SNP-biased group. In contrast, only the three-quarter of linkage and almost-linkage (one pair of markers not linked) can be detected in the STR-biased group. Only with more than 40 markers can the power of STR-biased panels reach 50% and more. In the unbiased group where the number of linked SNPs and linked STRs are equal, K presented more than 50% power when panel sizes were greater than 32 in half-linked panels and greater than 16 in three-quarter-linked panels; the power of X is not as strong as K, only greater than 50% when panel sizes are over 60 in half-linked panels and over 32 in three-quarter-linked panels. In another direction, the number of shared alleles (X) has a lower power than the number of heterozygous loci (K), but the type-I error is larger for K when the panel size is small (Fig. 3 and Additional file 6: Figure S6).

Afterwards, pure SNP panels were chosen tested to compare summary statistics and other methods. When the panels have more than 10 SNPs the power of both methods are larger than 50%, even in quarter-linked scenarios. For little-linked panels where only one pair of SNPs in linkage, summary statistics are able to detect with power of about 10–20% when panel size are smaller than twenty SNPs, which is much more powerful than pairwise LD. For quarter and half-linked panels, summary statistics presented their advantage with distinguished power for larger panels. In half-linked panels, summary statistics can detect linkage disequilibrium with power of approximately 75–80% when the sample size is larger than 20 and more than 40–50% in quarter-linked panels when the panel sizes are not smaller than 30. For panels with more than 30 SNPs, the power of summary statistics is larger than 40% and increases with the panel size. When there are more than 20 SNPs, the power is estimated to be 100% for fully linked loci, more than 50% for quarter-linked panels, and nearly 90% for half-linked panels (Additional file 7: Figure S7).

### Real data analysis

Furthermore, having been used in the tests on simulated panels, *mixIndependR* was applied to panels with real data. Based on the SNP + STR reference haplotype panel which was generated by imputing STRs to SNPs [11, 20], a mix panel of 2067 variants including 47 STRs and 2020 SNPs, with a threshold 0.2 for squared pairwise correlation -r^2^ (Details of variants selection in Methods). In PCA panel design, this is a threshold for “independent” variants. Refer to the output of $r^{2}$ table from plink, 0.36% of pairs are detected as pairwise LD ($r^{2} > 0.2$) in this panel though. Therefore, we expect detecting this level of linkage disequilibrium with K and X. With a sample size of 2504 individuals, *p* values of mutual independence test were calculated by K and X. Controlling the panel size for easy comparison in later research, panel size was defined as 100. In other words, 100 markers of the 2067 variants were selected randomly as a sample panel to test, and the process of choose and test was repeated in 1000 times.

As a results, in the 1000 trials 76.1% of *p* values of K and 19.0% of *p* values for X are smaller than 0.05, which indicates that the markers of this panel are not mutual independent. In contrast, the number of significant pairwise LDs calculated by *GDA* is 218 out of 4950 pairs of markers (100 markers were selected randomly in one sample), which is 4.40%. The large difference between the power of X compared to K might result from the fact that majority of the markers are SNPs, the heterozygosity of which data carries more information than number of sharing alleles. In contrast, the pairwise LDs between these markers gave a result of $r^{2}$ < 0.2, which is the selection rule for this panel. Our new approach with summary performed better than the traditional methods in the panels with little linkage in the real world. In addition, more panels with different threshold of $r^{2}$ have been designed and the comparison is presented in the section of Discussion.

## Discussion

**Fig. 4:**
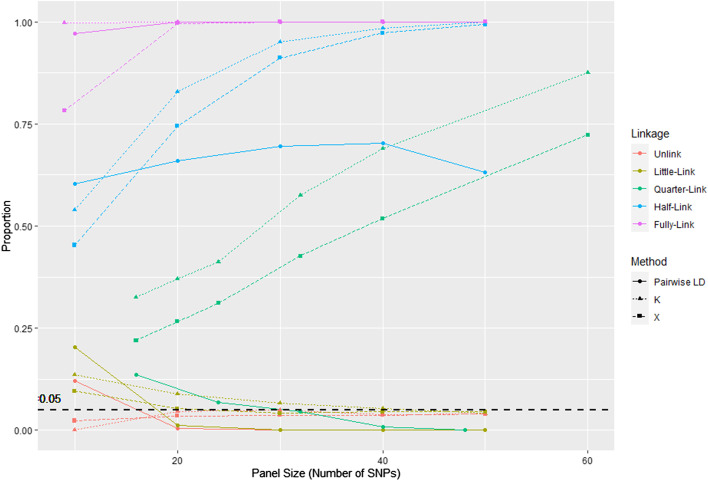
Power and significance level comparison between summary statistics and traditional LD test. Comparison among K, X, and pairwise LD calculated by R package genetics. K denotes number of heterozygous loci; X denotes number of shared alleles; Pairwise LD denotes the results from the function *LD* of package *genetics*

Compared to traditional LD tests (e.g., D′), the approaches presented herein appear to become more powerful as the size of the panel increases. In Fig. 4 presented the comparisons of power between summary statistics and pairwise LD on simulated pure SNP panels with different levels of linkage (Details of calculation in Methods). K and X showed shows more powerful with 10% than pairwise LD in half-linked panels and holds a stronger advantage in quarter-linked panels, where pairwise LD loss its power. For little-linked panels where there is only one pair of SNPs in linkage, summary statistics can detect linkage with about 20% power for small panel size.

**Fig. 5:**
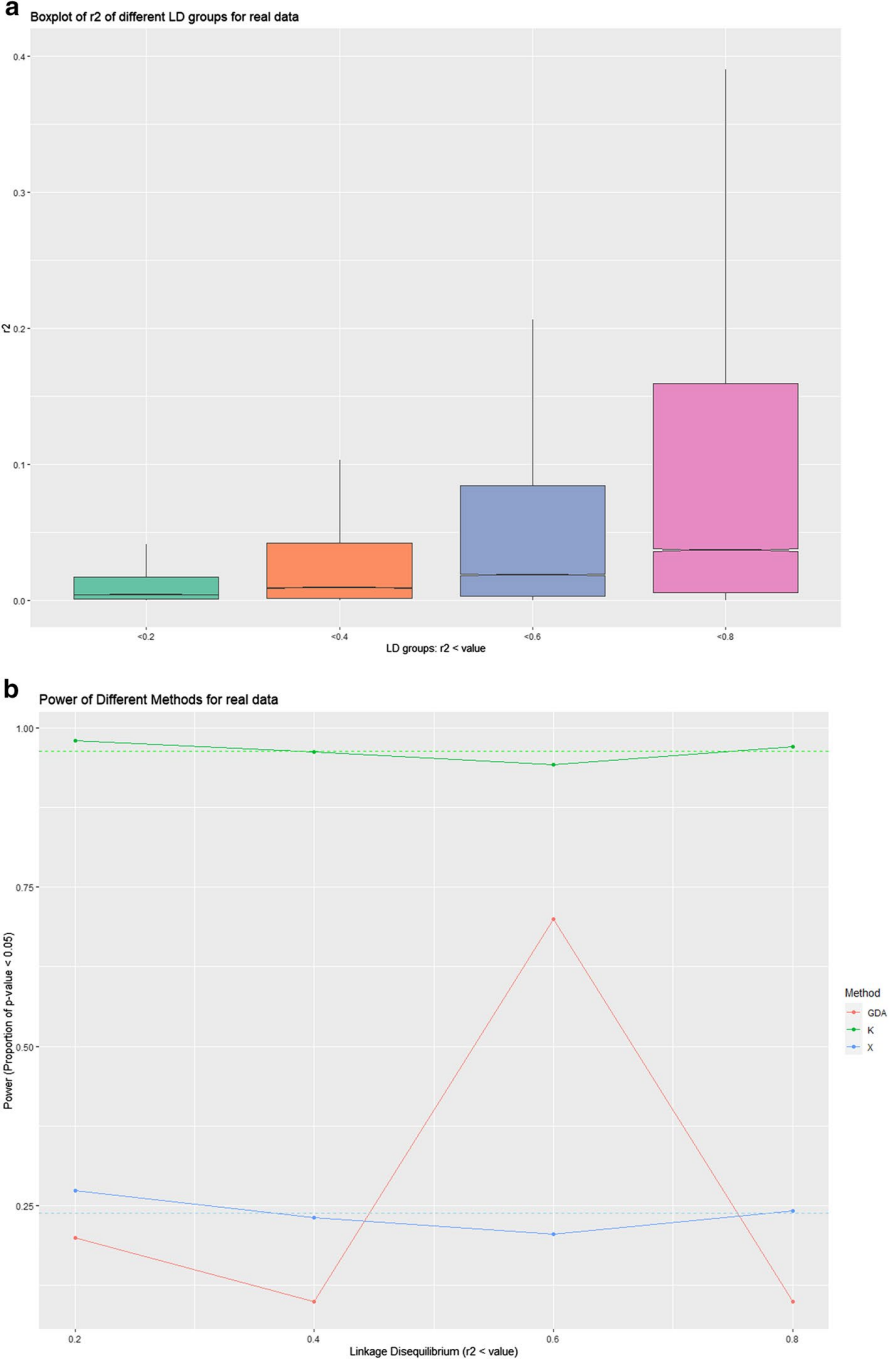
Comparison of K or X and pairwise LD in real data. Four panels designed from real dataset by variant pruning with threshold 0.2, 0.4, 0.6 and 0.8 are tested in pairwise LD and summary statistic K and X. In each panel, there are 2067, 3157, 4278 and 5754 variants, respectively. **a** is the boxplot excluding outliers of $r^{2}$ values for each panel. With the threshold increasing (x-axis denotes the groups), the boxplot lifts but major parts remain under 0.2. **b** is the power of pairwise LD by GDA, K, and X in multiple trials (10 trials for GDA, 1000 trials for K or X) for 100 random markers from each panel. On each new trial, the markers were re-selected. Y-axis shows the proportion of significant *p *values on each panel. The number of trials for GDA is small due to the time-consuming of this software. The power in this method might not be accurate. Average proportions for significant *p* values in method GDA is: 0.047 (0.2 group); 0.041 (0.4 group), 0.054 (0.6 group), 0.040 (0.8 group)

On the other hand, the tests of real data panels have also been completed and compared between the pairwise correlation and summary statistics- K and X. With the threshold for $$r^{2}$$ set in 4 groups: < 0.2, < 0.4, < 0.6 and < 0.8, four panels in Table 3 were designed. With the threshold lifting, the number of markers selected increase from 2067 to 5754, among which the number of STRs increases from 47 to 115. The percentage of variant pairs with significant LD in each group increases from 0.36 to 21.43%. Though the maximum of correlation was setting higher, the means of $$r^{2}$$ and 3rd quarters of $$r^{2}$$ remain below 0.2 (Table 3 and Fig. 5a). Using the software *GDA*, a set of *p*-values for pairwise LD were calculated (Fig. 5b). After the Bonferroni correction, about 3–5% pairs of variants are found significant dependent in each test of a panel with 100 variants. Holding the panel size as 100 constant, 10 times of analysis were repeated with different 100 variants which were chosen randomly from each group, and the power of method *GDA* has been assessed using the formula explained in Methods. Due to the few repeats of experiments, most of the power is below 20%. Though 7 of the 10 repeats at the group of 0.6 have been found as significance, the average of proportion of significant *p* values after Bonferroni correction is 0.054. The seven proportions defined as dependency panel are range of 0.05 and 0.06. This result might be explained by not enough times of experiments. More trials can be tested, but this method is time-consuming as one single calculation for pairwise LD of a 100-variant-panel needs 1 h and 8 min, without the time of generating and formatting samples. However, in the tests using K and X, linkage disequilibrium was detected. Controlling panel size as 100 markers, the proportion of significant *p* values (< 0.05) is 96.35% on average for K and 23.80% for X. Across four groups with different threshold of $$r^{2}$$ and total panel size, the power of K or X fluctuate in a small scale ($$sd_{K} = 0.016. sd_{X} = 0.029)$$. The reason why K shows a much stronger power than X also lies in the large proportion of SNPs in panels. Also, K or X can conduct a single test in 100 times with different random samples selected each time in only 12 and a half minutes for panels with 100 markers.

**Table 3:** Summary of panels designed from real data

LD (r2 threshold)	< 0.2	< 0.4	< 0.6	< 0.8
*Number of different type of markers*				
Number of Markers	2067	3157	4278	5754
Number of STRs	47	64	83	115
Number of SNPs	2020	3093	4195	5639
*Summary of r2*				
Min	0.000	0.000	0.000	0.000
1st Qu	0.000	0.002	0.003	0.006
Median	0.000	0.009	0.018	0.037
Mean	0.020	0.042	0.074	0.124
3rd Qu	0.020	0.042	0.084	0.159
Max	0.990	0.991	0.991	0.991

This is the summary for panels designed out of real data. On the chromosome 22, 4 mixed panels have been generated from the SNP-STR reference haplotype panel of Gymrek’s lab [20]. The pruning was completed on plink/1.90 with threshold of pairwise correlation 0.2, 0.4, 0.6 and 0.8, with the code “--indep-pairwise”. The second part of Table 3 presents the summary of $r^{2}$ values for each panel. The lists of $r^{2}$ were calculated and exported with the code “ –r2 –ld-window-r2 0”.

Therefore, both in simulation and real data, this approach presented a stronger power on detecting linkage disequilibrium in a manner of testing the linkage more than pairwise of a whole panel, regardless of the types of genetic markers.

## Conclusions

The R package *mixIndependR* is available on the Comprehensive R Archive Network (CRAN) at: https://cran.r-project.org/web/packages/mixIndependR/index.html [21] and the development version can be found on Github: https://github.com/ice4prince/mixIndependR.git [22]. This package contributes to the LD test of mixed panels with different types of genetic markers. It permits several new approaches to estimating LD, with the described method permitting LD estimation between heterogeneous marker types (e.g., SNPs and STRs). Instead of pairwise LD, the method tests mutual independence across all the markers of one panel. With new test statistics, this approach improved the power of dependency testing, and succeeded in testing the overall linkage disequilibrium across all sites simultaneously.

## Methods

### Data representations

In *mixIndpendR*, dataset to import can be an “. xslx” “.csv” or “vcf” file, with the marker names in the first row, and sample ID in the first column like the example (Table 4). “vcf” file can be imported with the function *read_vcf_gt*. Allele files where alleles are separated in two different cells needs a conversion by function “*makeGenotypes*” in *genetics*[23, 24].

**Table 4:** Example of imported data

Sample ID	STR1	SNP1
1	12|12	A|A
2	13|14	T|T
3	13|13	A|T
4	14|15	A|T
5	15|13	T|A
6	13|14	A|T
7	14|3	A|A
8	12|2	T|A
9	14|14	T|T
10	15|15	A|T

Each row denotes each individual sample; each column denotes each marker. The format of csv file can be imported directly by “read.csv” and the vcf file can be imported with the function “*read_vcf_gt*” in this package. The allele separator is not restricted to “|”. It can be specified in the following functions

We simulated unlinked and linked data under the Wright-Fisher neutral model using the software *ms* [18]; the microsatellite data were converted by *microsat* [19] from *ms* format. For data simulation, we chose the average mutation rate ($\mu$) 1.3 × 10^–3^ [25] and an effective population (N) size of 3100, the latter of which was estimated from linkage disequilibrium [26]. Thus, to simulate the unlinked STR data the mutation parameter $\theta { }\left( {\theta = 4{\text{N}}\mu { }} \right)$ is equal to 16.12. In contrast, to simulate SNPs a fixed number of segregating sites was simulated for both unlinked data and linked data.

Real data was downloaded and filtered from the reference panel of Dr. Saini et al. [11] using *plink 1.90* [16, 17]. Chromosome 22 was chosen so that fewer variants needs to import, and less time will be spent. Before selecting for different levels of pairwise dependence, variants and samples with more than 1% missing data, with minor allele frequency lower than 0.1, and those who fail in Hardy–Weinberg test at 5%, and with Mendel error rate higher than 0.1 at family level or higher than 0.2 for SNPs, had been filtered out. As to the different levels of linkage panels, selection rules were set using the pairwise correlation-r^2^. Four groups were formed with pairwise correlation (window size 50 kb and step size 10 variants) < 0.2 (2067 variants), < 0.4 (3157 variants), < 0.6 (4278 variants) and < 0.8 (5754 variants), and in each panel there are 47 STRs, 64 STRs, 83 STRs, and 115 STRs, respectively. The LD significant pairs of variants in these groups make up 0.36%, 5.71%, 12.21%, and 21.43% among all variants in each panel, respectively. Furthermore, the pairwise r^2^ values for each group were exported by *plink*.

The example dataset attached in the package “*mixexample*” was filtered from chromosome 4 with the same selection rule but with a threshold 0.1 for $r^{2}$. Among the panel of 7413 variants, 100 markers were selected randomly to make up the example dataset.

## Functionalities

**Fig. 6:**
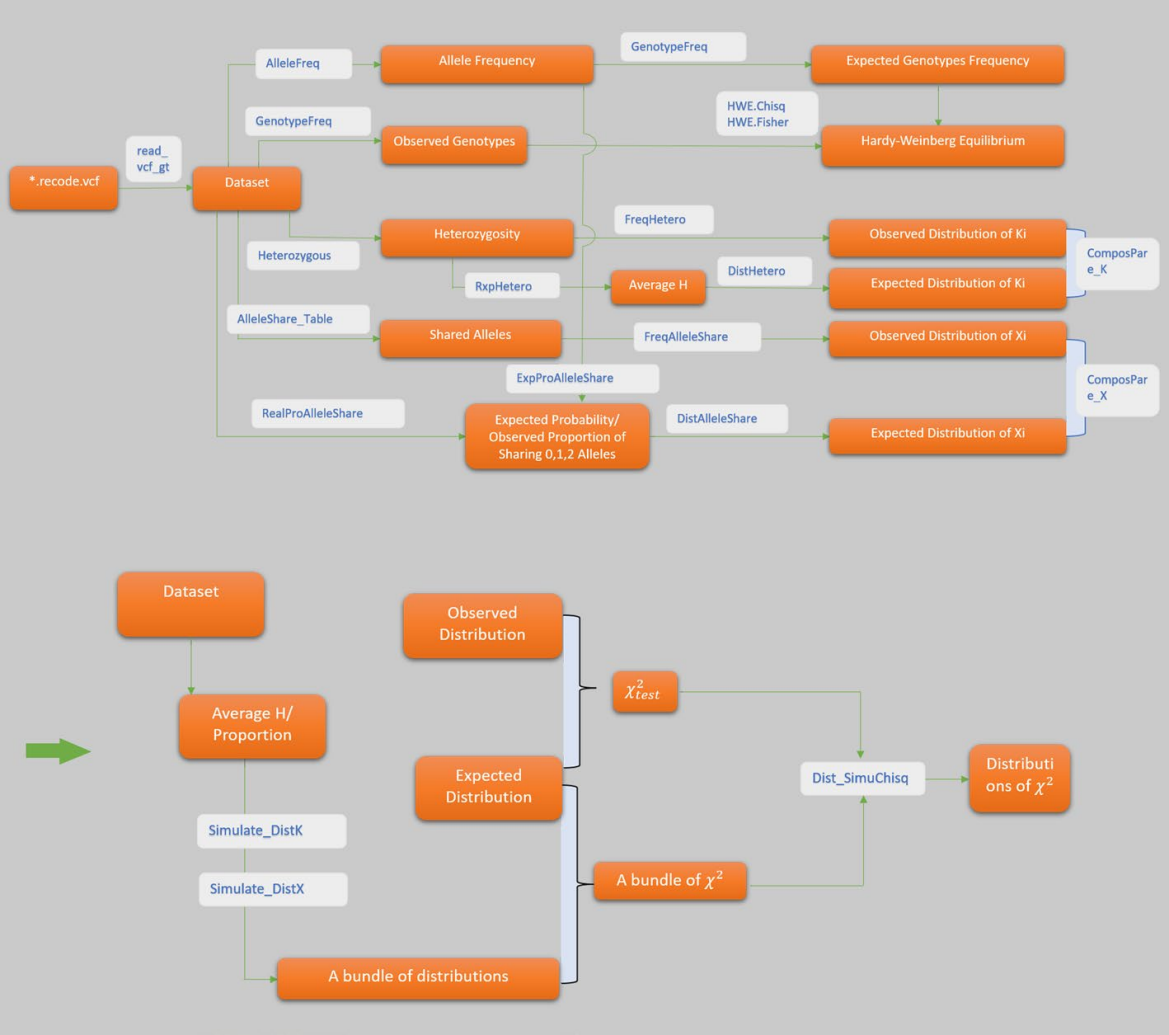
Pipeline of *mixIndependR*. Functions are presented in the grey boxes, and the results are in dark red boxes. The same function in different paths use different logic parameters. Crossed paths share input parameters

The package *mixIndependR* is made up of two main sections—basic genetic parameters calculation and mutual independence testing. Parameters obtained in the first part can be used in the second section. The software pipeline presented a structure of all the functions (Fig. 6).

### Basic genetics

The first part of this package was developed to calculate the basic genetic parameters of a mixed or non-mixed panel, including the allele frequencies *(AlleleFreq),* the genotype frequencies or expected genotype probabilities *(GenotypeFreq)*, and the Hardy–Weinberg Equilibrium *(HWE.Chisq)* [8, 27]. Different from other packages or software, these functions for basic genetic parameters can ignore the types of genetic markers and do not need to input the list of marker names, but the separator between alleles needs to be specified.

### Mutual independence

#### Summary statistics design

In traditional statistics, non-random associations were tested from pairs. However, mutual independence also included other subsets, like triplets and quartets. As the number of subsets increases, large quantities of free parameters will be generated. For example, for independence of two loci, there would be 3 free parameters if the heterozygosity were chosen as the tested statistics. But for 5 loci where the number of pair comes to 32, there would be 2^5^ − 1 = 31 free parameters in only pairwise independence testing, not to mention more subsets like triplets and quartets. When tens of loci were imported, the parameters would increase to thousands, where even the sample size might not be that large sometimes.

In order to break the large barrier that the number of free parameters might exceed the relatively small number of observations/subjects for the traditional statistic method when the sample size is not big enough, to avoid numerous calculations, and to reduce time consumption, two summary statistics were designed—the number of heterozygous Loci (K) and the number of shared alleles (X) [10].

Under the assumption of mutual independence, the distribution of these summary statistics follows a restricted multinomial distribution. By comparing the observed and expected distribution, the hypothesis for mutual independence could be tested [10].

For the number of heterozygous loci (K), the distribution follows an appropriate binomial distribution of 0 and 1, with the fixed but different probabilities on each trial. Similarly, the distribution of the number of shared alleles(X) follows a “skewed” multinomial distribution of 0, 1, and 2, where the probabilities are specified on each trial.

#### The number of heterozygous loci (K)

The number of heterozygous loci (K) is the sum of the number of heterozygous loci for each individual, which was denoted as

$$\begin{aligned} & K = K_{1} + K_{2} +K_{3} + \cdots K_{i} + \cdots + K_{m} \quad where \;m\;is\;the\;number\;of\;loci; \\ & K_{i} \;denotes\;the\;status\;of\;heterozygosity\;of\;the\;i_{th} \;loci,\ \\& 0\;as\;homozygous, and\;1\;as\;heterozygous; \\ & when\;m\;is\;the\;number\;of\;loci,\;K\;belongs\;to\;\left( {0, m} \right) \\ \end{aligned}$$

With the dataset imported*,* a table for heterozygosity was built by *Heterozygous.* This function exported a table of 0 and 1, where 0 denoted homozygous and 1 denoted heterozygous, with each row for each individual and each column for each marker. With the table of heterozygous status, the function *FreqHetero* obtained the observed distribution of number of heterozygous loci for the targeted dataset.

The expected distribution was built according to a recurrence formula with a specially assigned “Start” and “End” [9]. For the first m-th loci, there were two possible scenarios to discover r heterozygous loci: 1. the last locus is homozygous and there were r heterozygous loci on the first (m − 1) loci; 2. the last locus is heterozygous and there were (r − 1) heterozygous loci on the first (m − 1) loci.

$$\begin{aligned} & P_{{{\text{x}} = {\text{r}}}}^{{\text{m}}} = P_{x = r}^{m - 1} \left( {1 - h_{m} } \right) + P_{x = r - 1}^{m - 1} h_{m} \\ & where\;h_{m} \;is\;the\;heterozygosity\;of\;the\;m\text{-}th\;locus \\ \end{aligned}$$

The vector of heterozygosity was calculated with function *RxpHetero* in case of Hardy–Weinberg Equilibrium or Disequilibrium with the logic parameter *HWE* true or false. The heterozygosity was saved into the vector *H* in the order of loci, and the expected distribution of K was calculated by *DistHetero(H).*

#### The number of shared alleles (X)

Similarly, the number of shared alleles (X) is the summation of the number of shared alleles on each locus obtained by comparing each two individuals.

$$\begin{aligned} & X = X_{1} + X_{2} + X_{3} + \cdots X_{i} \cdots + X_{m} \quad {\text{where}}\;{\text{m}}\;{\text{is}}\;{\text{number}}\;{\text{of}}\;{\text{loci}} \\ & X_{{\text{i}}} \;{\text{denotes}}\;{\text{the}}\;{\text{number}}\;{\text{of}}\;{\text{shared}}\;{\text{alleles}}\;{\text{on}}\;{\text{the}}\;i_{th} \;{\text{locus}};\;{\text{belongs}}\;{\text{to}}\;\left( {0,1,2} \right) \\ & X\;{\text{belongs}}\;{\text{to}}\;\left( {0,{ }2{\text{m}}} \right){ } \\ \end{aligned}$$

A table of $X_{{\text{i}}}$ was built by *AlleleShare,* where a logic parameter “*replacement*” defining the type of “pick pairs” needs to be clarified as *TRUE* for pairs with replacement or *FALSE* for pairs without replacement.

The observed distribution of $X_{i}$ was built by *FreqAlleleShare* from the result of *AlleleShare.* As for the expected distribution, the probability for sharing 0, 1, or 2 alleles on each locus was needed. According to Weir, the expected probabilities of sharing 0, 1, or 2 alleles for two unrelated individuals could be obtained by *ExpProAlleleShare,* with the allele frequency table from *AlleleFreq*. On the other hand, for non-ideal samples, the real proportions of sharing alleles were also suitable to use, which could be calculated by *RealProAlleleShare.* With the probability table, an expected distribution of $X_{i}$ was built by *DistAlleleShare* by the principles of multinomial distribution

$$\begin{aligned} & P_{x = r}^{m} = P_{x = r}^{m - 1} *p_{0}^{m} + P_{x = r - 1}^{m - 1} *p_{1}^{m} + P_{r - 2}^{m - 1} *p_{2}^{m} \\ & where\;p_{k}^{m} \;denotes\;the\;probability\;of\;have\;k\;shared\;alleles\;on\;the\;m\text{-}th\;locus \\ \end{aligned}$$

#### Significance test

With the distributions built, two functions were designed for data visualization: *ComposPare_K* and *ComposPare_X*. These functions generated expected frequencies from the probabilities and converted the dataset into a form suitable to *ggplot2* [28] or *plot* in R.

The null hypothesis was that all markers, regardless of which type, are independent from each other. To test the null hypothesis, making comparisons between observed and expected distribution was required. Generally, a multinomial distribution has a fixed probability for each trial. However, in genetics, we cannot predict the distribution of heterozygosity or probability of sharing alleles for an unknown panel under testing. Thus, there were no perfect statistical methods designed for this special problem at this moment.

Despite the lack of existed methodology, with the fixed probability, it was not difficult to find the distribution of the test statistics by large sample simulation.

In this research, chi square value was chosen as the test statistic since the test was for fit of goodness and the distribution was similar to multinomial distribution. To ensure a good approximation for the chi square distribution, the expected numbers of each category were restricted to no less than five. Functions *Simulate_DistK* and *Simulate_DistX* built the simulation for K and X, respectively. For each new panel, each new bundle of simulation needs to be built. Via the basic equation of calculating the Chi-square value, a set of Chi-square values were generated by function *Dist_SimuChisq.* With the set of Chi-square values, a plot of cumulative distribution of $\chi^{2}$ were drawn clearly, from which the critical value of Chi-square statistic was specified.

### Comparison with other software

The R package *genetics* provides a function *LD* to test the linkage disequilibrium for all possible pairs of loci in one panel. This function can output D, D′, Pearson’s correlation coefficient, Chi-square statistic for linkage equilibrium (D = D′ = 0) and the *p* value of Chi-square test for independence. Using the unlinked data and linked data simulated above, a distribution of the proportion of *p* values smaller than significance level after Bonferroni correction can be obtained. The null hypothesis is that there is no significant LD among all the pairs for unlinked panel, which is $\Pr \left( {p \text{-}values < \frac{0.05}{{number\,of\,pairs}}} \right) = 0$. Under the null hypothesis, even though the markers were simulated on different chromosomes and there should be no association between any pair of them, some random associations may still be generated. In this program, no more than 5% random associations among the 1000 runs was allowed. In other words, if the percentage of significant *p* values is larger than 0.05, the null hypothesis would be rejected.

## Supplementary Information

**Additional file 1: Figure S1.** Distribution of number of heterozygous loci (K) for the example dataset. The X-axis is the number of shared alleles from 0 to 100, and the Y-axis is the observed density or expected probability of each K. The red bar is the expected distribution, and the green bar denotes the observed distribution. The red line is the expected spline for probability of K.

**Additional file 2: Figure S2.** Distribution of number of shared alleles (X) for the example dataset. The X-axis is the number of shared alleles from 0 to 200, and the Y-axis is the observed density or expected probability of each X. The red bar is the expected distribution, and the green bar denotes the observed distribution. The red line is the expected spline for probability of X.

**Additional file 3: Figure S3.** Cumulative probability function of Chi-square values for number of heterozygous loci (K) of the example dataset. The Y-axis is the cumulative probability (1- p-value) for the chi-square value at X-axis. In this example, the tested chi-square value of K is 77.417 (blue line), with a cumulative probability of 0.924. In contrast, the critical value for p-value = 0.05 (cumulative probability = 0.95) is 112.9 (red line).

**Additional file 4: Figure S4.** Cumulative probability function of Chi-square values for number of shared alleles (X) of the example dataset. The Y-axis is the cumulative probability (1- p-value) for the chi-square value at X-axis. In this example, the tested chi-square value of X is 62.299 (blue line), with a cumulative probability of 0.778. In contrast, the critical value for p-value = 0.05 (cumulative probability = 0.95) is 115 (red line).

**Additional file 5: Figure S5.** Summary of power tests for simulated data at different linkage levels for K and X- comparison between ‘*Bias*’. The X-axis denotes the number of markers in each panel, and the Y-axis is the proportion of significant cases whose p-value is smaller than 0.05. The colors present different categories of *‘Bias’*- when ‘*Bias*’ = Both, the types of linked markers are both SNPs and STRs with equal numbers; when ‘*Bias*’ = SNP, the most or all linked markers are SNPs; when ‘*Bias*’ = STR, the most or all linked markers are STRs. In Fig. 5, the left plots are those for number of heterozygous loci (K) and the right plots are number of shared alleles (X). From the top to the bottom, the levels of linkage increase from “OnePair-Linked” to “Almost-Linked”. In the “Onepair/Little-Linked” level, only one pair of markers are linked in each panel; in the “HalfQuarter-Linked” level, 12.5% SNPs are linked; in the “Quarter-Linked” level, 25% SNPs are linked; in the “ThreeQuarter-Linked” level, 75% SNPs are linked; in the “Almost-Linked” level, all except one pair of markers are linked.

**Additional file 6: Figure S6.** Comparison of Power for mixed simulated data (‘*Bias*’ = Both) between number of heterozygous loci (K) and number of shared alleles (X). The X-axis denotes the number of markers in each panel, and the Y-axis is the proportion of significant cases whose p-value is smaller than 0.05. “Linkage on Both” means half of the linked markers are SNPs and the other linked markers are STRs. The solid lines are presented the power trends of K, and the dotted lines are the power trends of X. Different colors are different linkage levels: red lines are half-linked panels (50% markers are linked); green lines are Quarter-Linked panels (25% markers are linked); blue lines are ThreeQuarter-Linked panels (75% markers are linked).

**Additional file 7: Figure S7.** Power and significant level of K and X for simulated SNP panels at different linkage levels. The proportions of significant cases out of 1000 trials along different panel size for simulated SNP panels at different linkage levels. In the “Unlinked” level, all markers in each panel are unlinked; in the “Onepair/Little-Linked” level, only one pair of markers are linked in each panel; in the “HalfQuarter-Linked” level, 12.5% SNPs are linked; in the “Quarter-Linked” level, 25% SNPs are linked; in the “ThreeQuarter-Linked” level, 75% SNPs are linked; in the “Almost-Linked” level, all except one pair of markers are linked; in the “Fully-Linked” level, all markers in each panel are linked. The axis “Panel Size” denotes the number of the SNPs in each panel.

**Additional file 8: Table S1.** Genotypes of example dataset—‘*mixexample*’. The raw genotypes of the example data. Each row denotes each individual and each column denotes each marker.

**Additional file 9: Table S2.** Table of allele frequency (expected) for example dataset- ‘*mixexample*’. Each column denotes each marker. The first column lists all alleles appeared at all loci-0, 1, 2, 3, 4, and 5. There is the probability of the allele of this row at the locus of this column.

**Additional file 10: Table S3.** Table of heterozygosity for example dataset- ‘*mixexample*’. In each cell, the status of heterozygosity -0 (homozygous) or 1 (heterozygous), for the individual of this row at this locus is presented. Column names are markers’ names, and the row names are individuals.

**Additional file 11: Table S4.** Table of shared alleles for example dataset- ‘*mixexample*’. In each cell, the number of shared alleles -0 or 1 or 2, for the pair of individuals of this row at this locus is presented. Column names are markers’ names, and the row names are pairs of individuals.

**Additional file 12: Table S5.** Simulations for expected number of heterozygous loci (K) for the example dataset- ‘mixexample’. The first row (also the column names) is the number of heterozygous loci (K) from 0 to 100; and the first column is the index of simulations 1 to 500. Each row denotes each simulation and shows one distribution of K.

**Additional file 13: Table S6.** Simulations for expected number of shared alleles (X) for the example dataset- ‘mixexample’. The first row (also the column names) is the number of shared alleles from 0 to 200; and the first column is the index of simulations- 1 to 500. Each row denotes each simulation and shows one distribution of X.

**Additional file 14: Table S7.** Summary of power test for simulated mix panels. Full version of Table 2. The first sheet includes all designed panels; and sheet 2 ~ 4 filtered on variable ‘Bias’. Sheet 2 presents the panels when half of linked markers are SNPs and the other half of linked markers are STRs; Sheet 3 presents the panels with more linked SNPs than linked STRs; Sheet 4 presents the panels with more linked STRs than linked SNPs.

## Data Availability

The package is available on both CRAN: CRAN—Package mixIndependR (r-project.org) and GitHub(development version): ice4prince/mixIndependR: R Package mixIndependR (github.com) and all the data used in this project are available in “Additional Data and Material” section of GitHub: mixIndependR/Additional Data and Material at main · ice4prince/mixIndependR (github.com).

## Abbreviations

SNP: Single nucleotide polymorphism
InDel: Insertions and deletions
STR: Short tandem repeats
LD: Linkage disequilibrium
K: The number of heterozygous loci
X: The number of shared alleles
CRAN: The R package mixIndependR*,* is available on the Comprehensive R Archive Network
CODIS: The combined DNA Index System
CEU: Northern and Western Europe
JPT: Japanese from Tokyo
HCB: Han Chinese from Beijing

## References

[1] Butler JM, Coble MD, Vallone PM (2007). STRs vs. SNPs: thoughts on the future of forensic DNA testing. Forensic Sci Med Pathol.

[2] Wei T, Liao F, Wang Y, Pan C, Xiao C, Huang D (2018). A novel multiplex assay of SNP-STR markers for forensic purpose. PLoS ONE.

[3] Wang L, He W, Mao J, Wang H, Jin B, Luo HB, Liang WB, Zhang L (2015). Development of a SNP-STRs multiplex for forensic identification. Forensic Sci Int Genet Suppl Ser.

[4] Edge MD, Algee-Hewitt BFB, Pemberton TJ, Li JZ, Rosenberg NA (2017). Linkage disequilibrium matches forensic genetic records to disjoint genomic marker sets. Proc Natl Acad Sci USA.

[5] Schulze TG, Chen YS, Akula N, Hennessy K, Badner JA, McInnis MG, DePaulo JR, Schumacher J, Cichon S, Propping P (2002). Can long-range microsatellite data be used to predict short-range linkage disequilibrium?. Hum Mol Genet.

[6] Danecek P, Schiffels S, Durbin R. Multiallelic calling model in bcftools (-m). In: June; 2014.

[7] Zheng X, Levine D, Shen J, Gogarten SM, Laurie C, Weir BS (2012). A high-performance computing toolset for relatedness and principal component analysis of SNP data. Bioinformatics.

[8] Weir BS (1996). Genetic data analysis II: methods for discrete population genetic data.

[9] Chakraborty R (1981). The distribution of the number of heterozygous Loci in an individual in natural populations. Genetics.

[10] Chakraborty R, Stivers DN, Su B, Zhong Y, Budowle B (1999). The utility of short tandem repeat loci beyond human identification: implications for development of new DNA typing systems. Electrophoresis.

[11] Saini S, Mitra I, Mousavi N, Fotsing SF, Gymrek M (2018). A reference haplotype panel for genome-wide imputation of short tandem repeats. Nat Commun.

[12] Delourme R, Falentin C, Fomeju BF, Boillot M, Lassalle G, André I, Duarte J, Gauthier V, Lucante N, Marty A (2013). High-density SNP-based genetic map development and linkage disequilibrium assessment in *Brassica napus* L. BMC Genom.

[13] Li X, Han Y, Wei Y, Acharya A, Farmer AD, Ho J, Monteros MJ, Brummer EC (2014). Development of an alfalfa SNP array and its use to evaluate patterns of population structure and linkage disequilibrium. PLoS ONE.

[14] Danecek P, Auton A, Abecasis G, Albers CA, Banks E, DePristo MA, Handsaker RE, Lunter G, Marth GT, Sherry ST (2011). The variant call format and VCFtools. Bioinformatics.

[15] Weir BS (2004). Matching and partially-matching DNA profiles. J Forensic Sci.

[16] Purcell S, Neale B, Todd-Brown K, Thomas L, Ferreira MAR, Bender D, Maller J, Sklar P, de Bakker PIW, Daly MJ (2007). PLINK: a tool set for whole-genome association and population-based linkage analyses. Am J Hum Genet.

[17] PLINK 1.90. http://pngu.mgh.harvard.edu/purcell/plink/.

[18] Hudson RR (2002). Generating samples under a Wright-Fisher neutral model of genetic variation. Bioinformatics.

[19] Simulates the evolution of linked and unlinked microsatellites using the coalescent. https://github.com/mpcox/microsat.

[20] A reference haplotype panel for genome-wide imputation of short tandem repeats. http://gymreklab.com/2018/03/05/snpstr_imputation.html. 10.1038/s41467-018-06694-0 PMC6199332 30353011

[21] mixIndependR: genetics and independence testing of mixed genetic panels. https://cran.r-project.org/web/packages/mixIndependR/index.html.

[22] Song B. mixIndependR: genetics and independence testing of mixed genetic panels (Version v0.4.3). In: 2020, December 1.

[23] Gregorius HR (1980). The probability of losing an allele when diploid genotypes are sampled. Biometrics.

[24] genetics: population genetics. https://cran.r-project.org/web/packages/genetics/index.html.

[25] Dauber EM, Bär W, Klintschar M, Neuhuber F, Parson W, Glock B, Mayr WR (2003). Mutation rates at 23 different short tandem repeat loci. Int Congr Ser.

[26] Tenesa A, Navarro P, Hayes BJ, Duffy DL, Clarke GM, Goddard ME, Visscher PM (2007). Recent human effective population size estimated from linkage disequilibrium. Genome Res.

[27] Guo SW, Thompson EA (1992). Performing the exact test of Hardy–Weinberg proportion for multiple alleles. Biometrics.

[28] Wickham H (2016). ggplot2: elegant graphics for data analysis.

