# Supplementary figures and images for: mixIndependR: a R package for statistical independence testing of loci in database of multi-locus genotypes
Source: BMC Bioinformatics. 2021 Jan 6;22:12. doi: 10.1186/s12859-020-03945-0 (PMC7788837; doi:10.1186/s12859-020-03945-0)

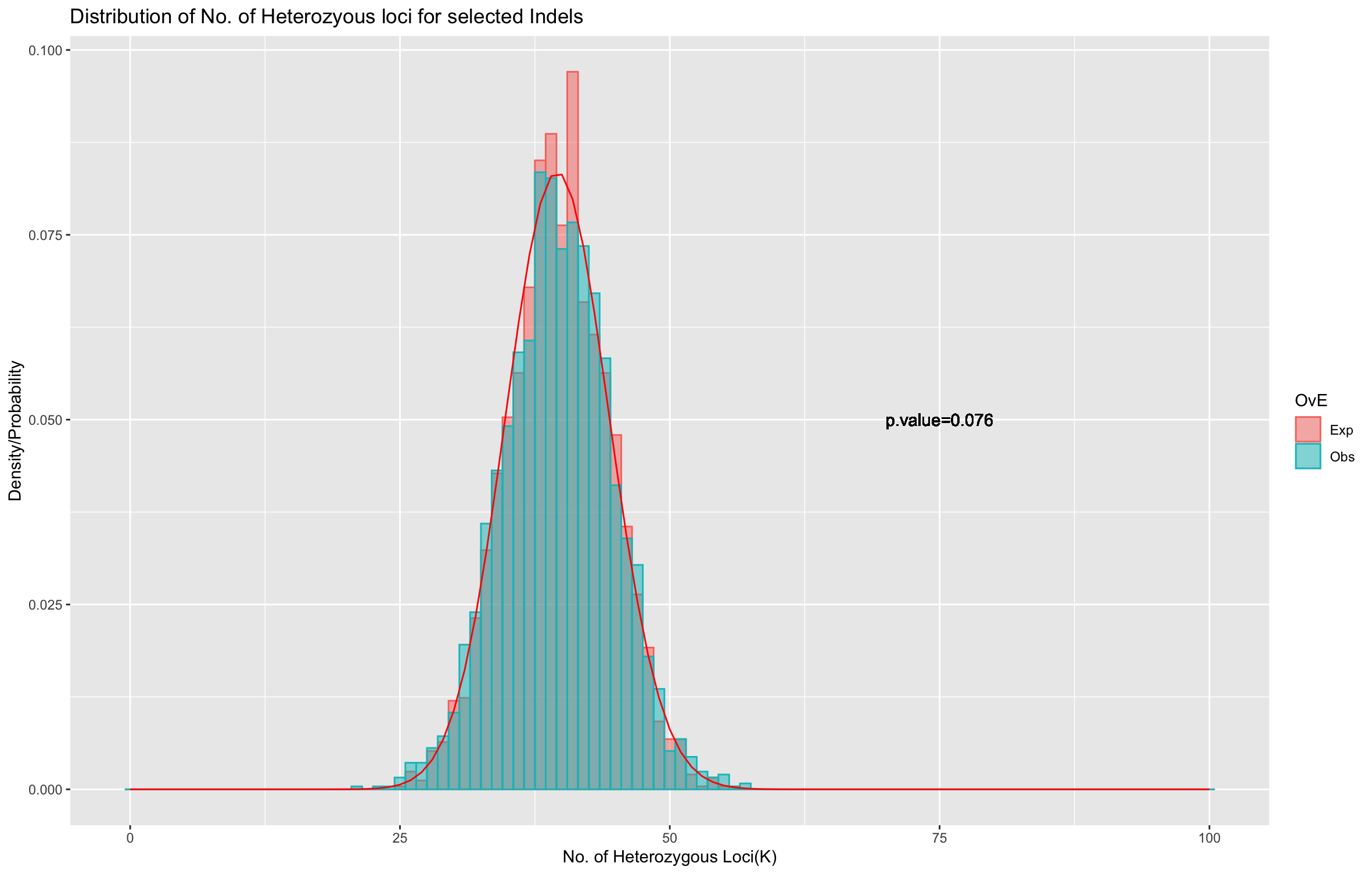

Supplement: Supplementary file 1 — Additional file 1: Figure S1. Distribution of number of heterozygous loci (K) for the example dataset. The X-axis is the number of shared alleles from 0 to 100, and the Y-axis is the observed density or expected probability of each K. The red bar is the expected distribution, and the green bar denotes the observed distribution. The red line is the expected spline for probability of K. [file 12859_2020_3945_MOESM1_ESM.png]

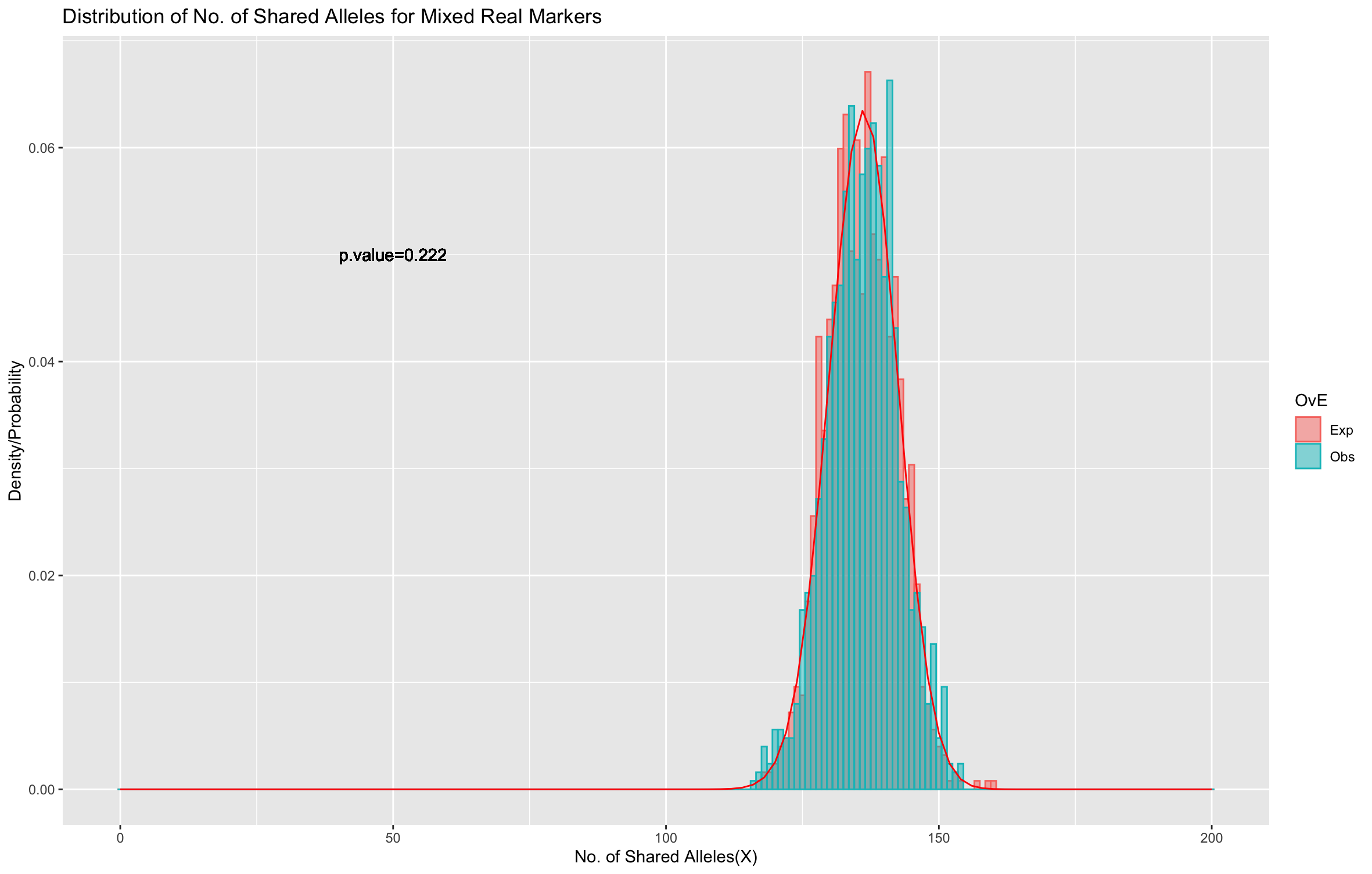

Supplement: Supplementary file 2 — Additional file 2: Figure S2. Distribution of number of shared alleles (X) for the example dataset. The X-axis is the number of shared alleles from 0 to 200, and the Y-axis is the observed density or expected probability of each X. The red bar is the expected distribution, and the green bar denotes the observed distribution. The red line is the expected spline for probability of X. [file 12859_2020_3945_MOESM2_ESM.png]

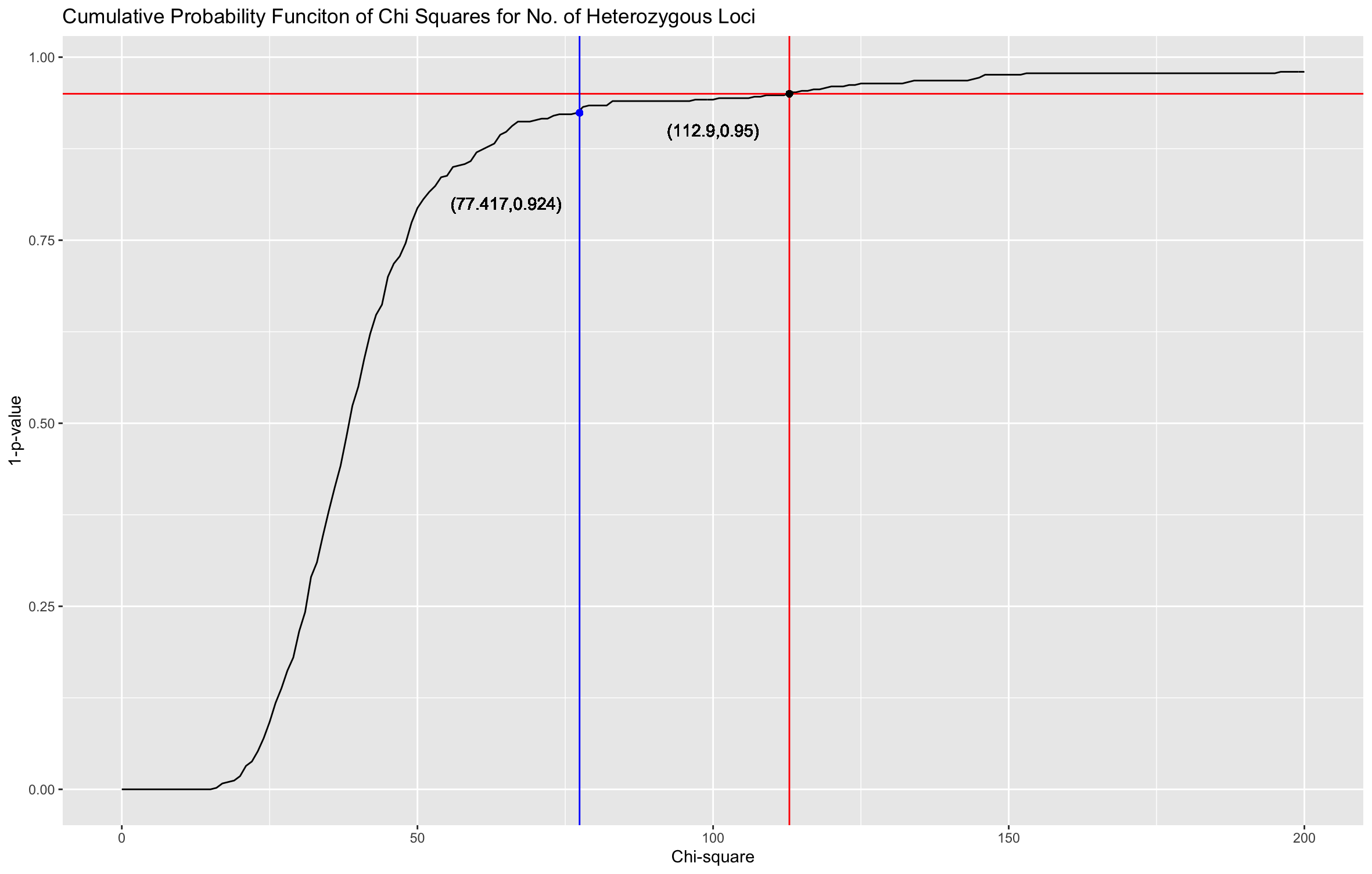

Supplement: Supplementary file 3 — Additional file 3: Figure S3. Cumulative probability function of Chi-square values for number of heterozygous loci (K) of the example dataset. The Y-axis is the cumulative probability (1- p-value) for the chi-square value at X-axis. In this example, the tested chi-square value of K is 77.417 (blue line), with a cumulative probability of 0.924. In contrast, the critical value for p-value = 0.05 (cumulative probability = 0.95) is 112.9 (red line). [file 12859_2020_3945_MOESM3_ESM.png]

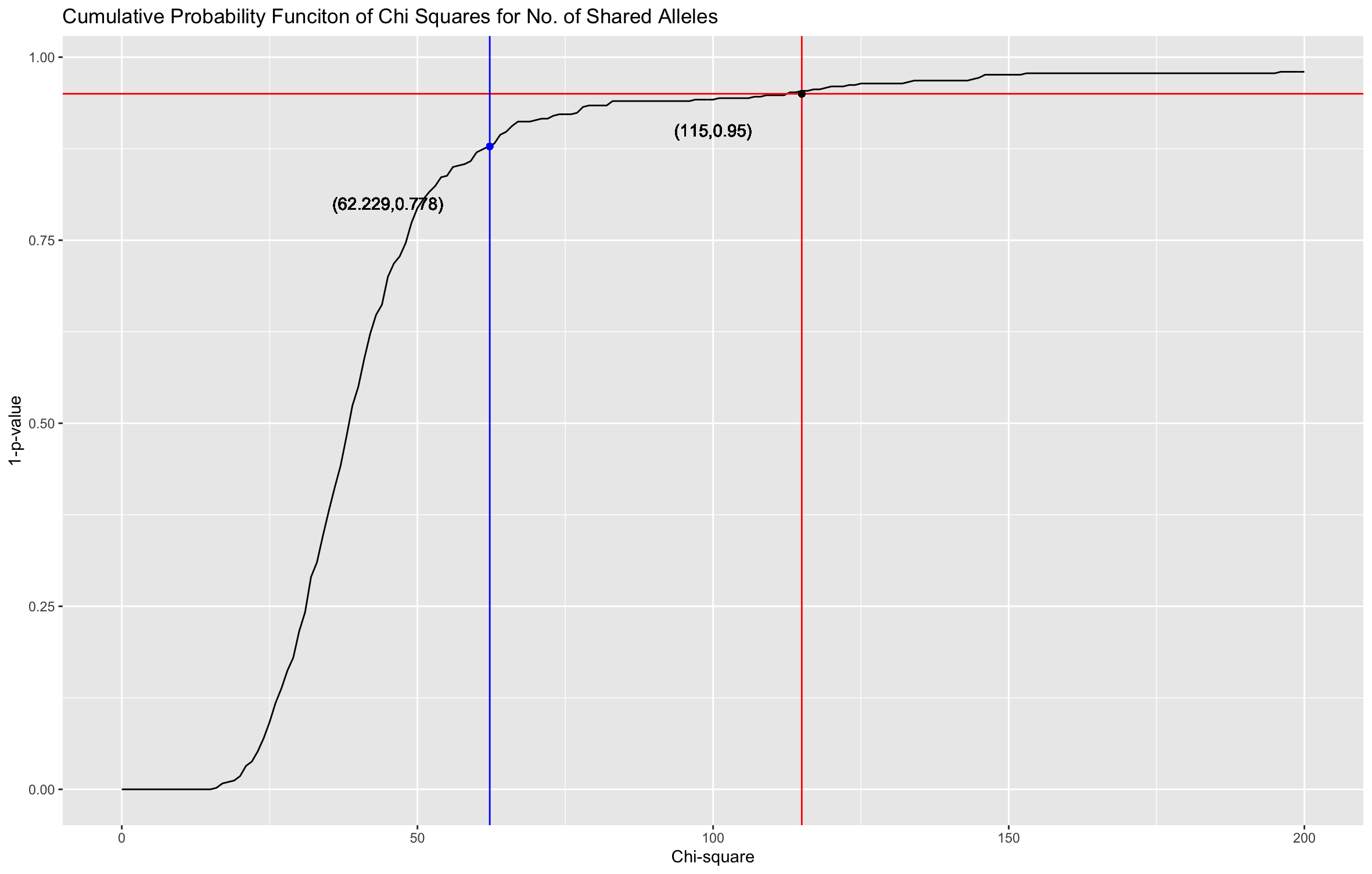

Supplement: Supplementary file 4 — Additional file 4: Figure S4. Cumulative probability function of Chi-square values for number of shared alleles (X) of the example dataset. The Y-axis is the cumulative probability (1- p-value) for the chi-square value at X-axis. In this example, the tested chi-square value of X is 62.299 (blue line), with a cumulative probability of 0.778. In contrast, the critical value for p-value = 0.05 (cumulative probability = 0.95) is 115 (red line). [file 12859_2020_3945_MOESM4_ESM.png]

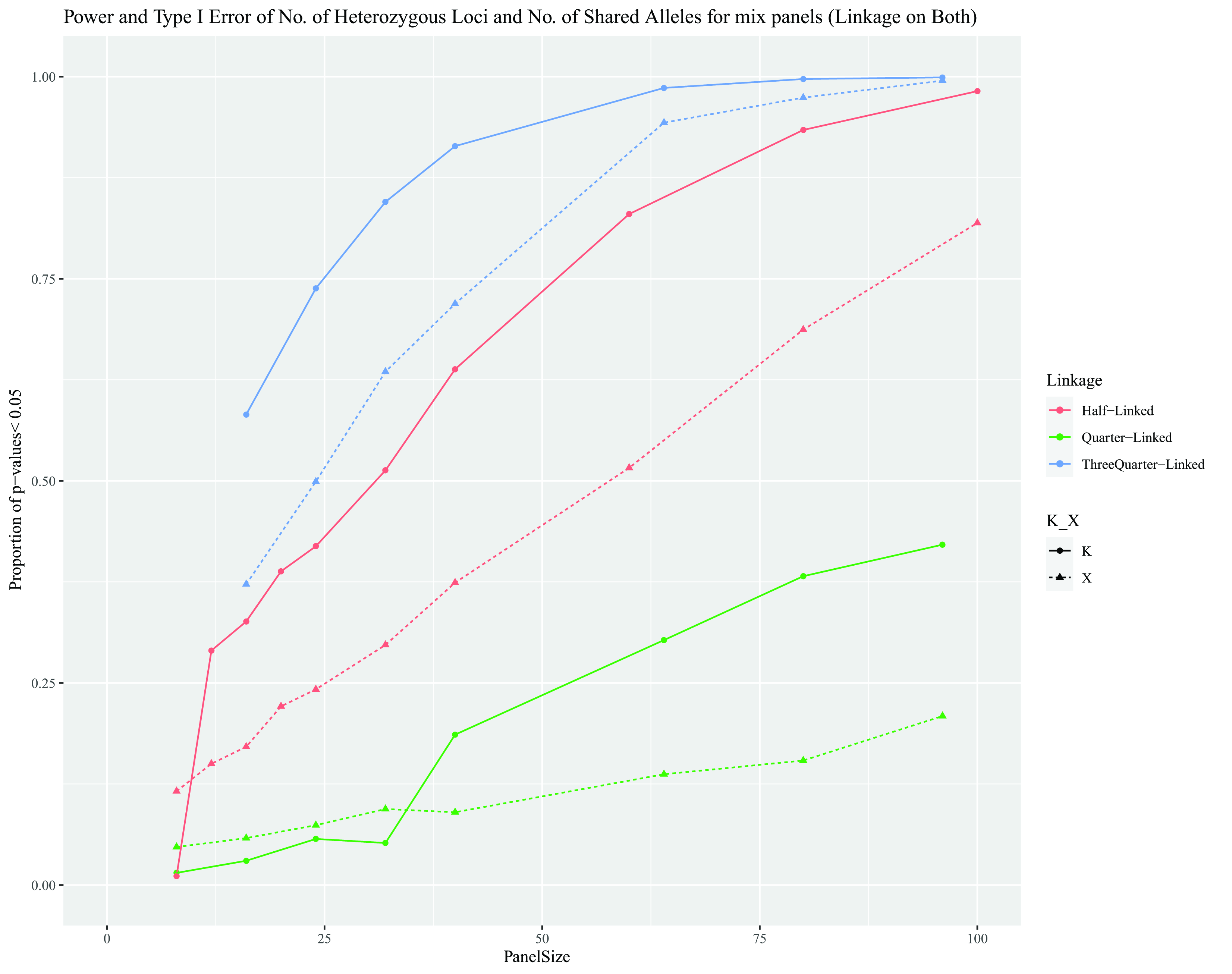

Supplement: Supplementary file 6 — Additional file 6: Figure S6. Comparison of Power for mixed simulated data (‘Bias’ = Both) between number of heterozygous loci (K) and number of shared alleles (X). The X-axis denotes the number of markers in each panel, and the Y-axis is the proportion of significant cases whose p-value is smaller than 0.05. “Linkage on Both” means half of the linked markers are SNPs and the other linked markers are STRs. The solid lines are presented the power trends of K, and the dotted lines are the power trends of X. Different colors are different linkage levels: red lines are half-linked panels (50% markers are linked); green lines are Quarter-Linked panels (25% markers are linked); blue lines are ThreeQuarter-Linked panels (75% markers are linked). [file 12859_2020_3945_MOESM6_ESM.tif]

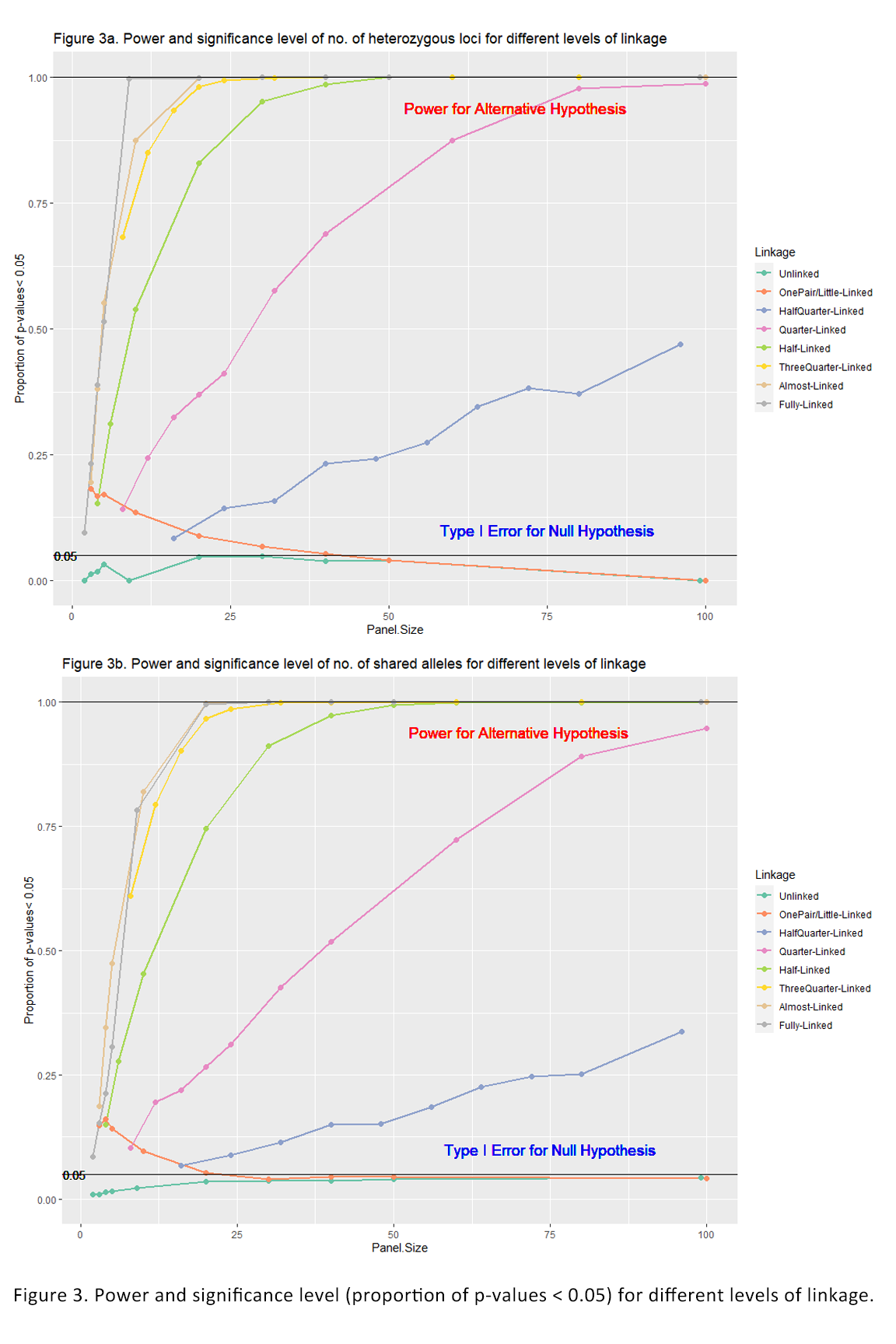

Supplement: Supplementary file 7 — Additional file 7: Figure S7. Power and significant level of K and X for simulated SNP panels at different linkage levels. The proportions of significant cases out of 1000 trials along different panel size for simulated SNP panels at different linkage levels. In the “Unlinked” level, all markers in each panel are unlinked; in the “Onepair/Little-Linked” level, only one pair of markers are linked in each panel; in the “HalfQuarter-Linked” level, 12.5% SNPs are linked; in the “Quarter-Linked” level, 25% SNPs are linked; in the “ThreeQuarter-Linked” level, 75% SNPs are linked; in the “Almost-Linked” level, all except one pair of markers are linked; in the “Fully-Linked” level, all markers in each panel are linked. The axis “Panel Size” denotes the number of the SNPs in each panel. [file 12859_2020_3945_MOESM7_ESM.png]
